# Supplementary material for: Research on complex dynamic behavior of automakers based on complex system theory
Source: PLoS One. 2024 Dec 6;19(12):e0314899. doi: 10.1371/journal.pone.0314899 (PMC11623478; doi:10.1371/journal.pone.0314899)
Supplement: Attachment — Submitted filename: Review.docx [file pone.0314899.s001.docx]

**Review of the Manuscript:**

**Title:** *Research on complex dynamic behavior of automakers based on complex system theory*

The article under review provides a comprehensive analysis of the dynamic behaviors of automakers – both those producing gas vehicles (GVs) and new energy vehicles (NEVs) – In the context of China’s dual credit policy (DC). Using complex system theory and a nonlinear dynamic system approach, the authors investigate the long-term production and emission strategies of NEV and GV automakers.

**Pros:**

- The article is well-organized, with a logical flow from the introduction through to the conclusions. It presents its methodology, findings, and implications in a clear manner, making it accessible to readers familiar with complex system theory.
- The focus on NEVs and GVs in the context of the dual credit policy is timely and relevant, given the global push toward sustainability and the rise of NEVs. This adds value to the research.
- The use of complex system theory and nonlinear dynamic systems to study the dynamics between NEV and GV automakers provides a novel approach to understanding the impact of dual credit policy on the auto industry. The study of stable regions, bifurcation, chaotic attractors, and the Largest Lyapunov exponent (LLE) is thorough.
- The article not only offers theoretical insights but also provides practical recommendations for policymakers and automakers regarding production strategies and emission reductions.
- The article also presents prospects for further research. In particular, they make an important emphasis on the fact that it makes sense to consider not just a duopoly of producers, but a triopoly. And that it is necessary to try to take into account other factors besides price or consumers’ low carbon preferences.
- The inclusion of mathematical modeling, particularly the long-term repeated game model, enhances the technical robustness of the study.

**Cons and recommendations:**

- Some key terms like the Lyapunov exponents and the Largest Lyapunov exponent could benefit from more detailed explanations for readers who may not be experts in nonlinear dynamic systems. Since you provide the reference about its usage for a dynamic game model for manufacturers and retailers, but immediately questions may arise regarding the general algorithm for calculating this indicator or its physical meaning. One may also wonder why Lyapunov exponents in this case will be better than the same Shannon entropy or fractal dimensionality. I think a couple of sentences with a formula and corresponding references to a more detailed explanation will not be superfluous.
- It is important to note that you repeatedly type the abbreviation LLE throughout the text, and yet you write “Largest Lyapunov exponent” in full throughout the text. In the introduction you write the full term “towards the development of new energy vehicles (NEVs).” with the abbreviation. However, in the next sentence, you again write the full term with the abbreviation “Given their potential to save energy and be more environment-friendly, new energy vehicles (NEVs) …”. Streamlining these sections could improve the overall readability of the manuscript.
- I think it makes sense to just leave captions (a), (b), (c), ... under each figure. and in each main caption we should write the values of parameters for, for example, bifurcation diagrams.
- It seems that there is a little mistake, in section 4.1.2, where you write “black dots indicate the carbon emission reduction $e$ of GV, and he pink line represents the LLE.”
- While the study is focused on China’s auto market, it would be helpful to include a discussion of how the findings might apply or differ in other countries or regions, given that the NEV transition is a global phenomenon. This should be considered in the next research.
- Equations should be followed by punctuation marks and the word “where” should not be capitalized after equations, as it goes along with them here.
- In the abstract, the sentence “with increase of production” should be revised to “with the increase in production.”
- Inconsistent spelling of terms like “behavior” and “behaviour”. In the abstract you write “we explored dynamic behaviours, …”

The article presents a novel and significant contribution to the study of the automotive industry’s transition to NEVs, with a robust theoretical foundation. With these revisions and minor corrections to the language itself, the article would be more widely applicable and accessible to both specialists and general readers. After considering the submitted comments, the article may be recommended for publication.
