# Supplementary material for: Research on complex dynamic behavior of automakers based on complex system theory
Source: PLoS One. 2024 Dec 6;19(12):e0314899. doi: 10.1371/journal.pone.0314899 (PMC11623478; doi:10.1371/journal.pone.0314899)
Supplement: Attachment — Submitted filename: Response to Reviewers.docx [file pone.0314899.s002.docx]

Dear Editor and reviewers of PLOS ONE：

I am writing to submit the revised version of our paper entitled **"Research on complex dynamic behavior of automakers based on complex system theory".** We would like to express our gratitude for the valuable time and suggestions you and the reviewers have devoted to our previous submission.

Thank you for your correspondence and the insightful feedback from the reviewers regarding our manuscript, "Research on complex dynamic behavior of automakers based on complex system theory". We greatly appreciate the value and constructive nature of their comments.

We have thoroughly reviewed each comment and have diligently incorporated the necessary revisions into our manuscript. Following your instructions, we have uploaded the revised manuscript with the changes highlighted in green to clearly indicate the additions made. Additionally, our responses to the reviewers' comments are also marked in green for easy reference.

We would like to express our gratitude once again for your letter and the clear and detailed feedback from the reviewer on our manuscript titled "Research on complex dynamic behavior of automakers based on complex system theory". We hope that these revisions address the reviewers' concerns and strengthen the contribution of our research to the field. We look forward to your further guidance and the opportunity to publish our work in your esteemed journal. In the remainder of this letter, we will discuss each of your comments and our corresponding responses individually.

**Comment 1:** *Some key terms like the Lyapunov exponents and the Largest Lyapunov exponent could benefit from more detailed explanations for readers who may not be experts in nonlinear dynamic systems. Since you provide the reference about its usage for a dynamic game model for manufacturers and retailers, but immediately questions may arise regarding the general algorithm for calculating this indicator or its physical meaning. One may also wonder why Lyapunov exponents in this case will be better than the same Shannon entropy or fractal dimensionality. I think a couple of sentences with a formula and corresponding references to a more detailed explanation will not be superfluous.*

**Response 1:** We appreciate your suggestion and apologize for the lack of clarity in our explanation of the Lyapunov exponent and the Largest Lyapunov exponent, especially for non-specialist readers. To address this, we have included a more detailed explanation in the revised manuscript, along with the relevant formulas, to enhance understanding of these concepts and their significance. The pertinent content is provided below for your quick reference.

*The Lyapunov exponent is an important quantitative tool for assessing the dynamic characteristics of a system, quantifying the average rate of convergence or divergence of neighboring trajectories in phase space over time. In particular, the LLE is crucial in revealing the chaotic nature of a system's long-term behavior. It more directly quantifies the sensitivity and chaotic characteristics of a system's dynamic actions. By observing whether the LLE is greater than zero, one can intuitively determine the system's state of dynamic chaos. A negative LLE indicates that the system is in a stable or period-doubling state; whereas an LLE of zero signifies a critical point in the system's state. Once the LLE is greater than zero, the system enters a chaotic state.* *Referring to the research of Zarepour et al. [52], the LLE calculation formula for discrete systems is defined as:*

$$LLE=\frac{1}{N}\log(\max\left|\lambda\prod_{i=0}^{N-1} J_{i}\right|),$$

*where* $\lambda$ *represents the eigenvalue of* $J_{1}$*, and* $J_{i}$ *is the Jacobian matrix calculated at each iteration.*

In response to your question, "Why is the Lyapunov exponent better in this situation than the Shannon entropy or fractal dimension?" we provide the following explanation.

Compared to Shannon entropy, the LLE is directly related to the system's dynamic behavior and sensitivity to initial conditions, while Shannon entropy primarily focuses on describing the system's uncertainty and information content. Fractal dimension characterizes the complexity and self-similarity of an object, whereas the LLE provides a direct measure of the system's degree of chaos. The LLE more intuitively reflects the chaotic characteristics of a system, particularly in dynamic game models, where it can more accurately predict how manufacturers' strategies evolve over time.

**Comment 2:** *It is important to note that you repeatedly type the abbreviation LLE throughout the text, and yet you write “Largest Lyapunov exponent” in full throughout the text. In the introduction you write the full term “towards the development of new energy vehicles (NEVs).” with the abbreviation. However, in the next sentence, you again write the full term with the abbreviation “Given their potential to save energy and be more environment-friendly, new energy vehicles (NEVs) …”. Streamlining these sections could improve the overall readability of the manuscript.*

**Response 2:** We appreciate your suggestion and sincerely apologize for any confusion regarding the use of abbreviations that you mentioned. In the revised manuscript, we have made adjustments to the terms "Largest Lyapunov exponent (LLE)" and "New Energy Vehicles (NEVs)" to ensure clarity and conciseness.

(1) When the "Largest Lyapunov exponent (LLE)" first appears in the introduction, we use its full name, and thereafter we will consistently use the abbreviation "LLE" to maintain terminology consistency.

(2) For the term "New Energy Vehicle (NEV)," we will introduce it with its full name the first time it appears in the introduction, and thereafter we will consistently use the abbreviation "NEV" to avoid unnecessary repetition and enhance the smoothness of the text.

**Comment 3:** *I think it makes sense to just leave captions (a), (b), (c), ... under each figure. and in each main caption we should write the values of parameters for, for example, bifurcation diagrams.*

**Response 3:** We appreciate your suggestion and are grateful for your constructive feedback. Your insights will significantly enhance the clarity and readability of our paper. We have adjusted the formatting in the text according to your recommendations, retaining only the labels for parameters, such as (a), (b), (c), etc., under each main heading, while ensuring that the specific values or descriptions of these parameters are clearly stated within each main heading. The relevant content is provided below for your quick reference.

|  |  |
| --- | --- |
| (a) | (b) |
|  | |
| (c) | |
| Figure 2. Bifurcation and LLE diagrams for system (3) concerning the adjustment speed of NEV production under the conditions $\alpha_{2}=0.1$ and $\alpha_{3}=0.2$. ((a) Bifurcation diagram with $\alpha_{1}\in[0,0.18]$. (b) A detailed bifurcation diagram with $\alpha_{1}\in$[0.156, 0.17]. (c) LLE diagram with $\alpha_{1}$ spanning the range [0, 0.18].) | |

**Comment 4:** *It seems that there is a little mistake, in section 4.1.2, where you write “black dots indicate the carbon emission reduction of GV, and he pink line represents the LLE.”*

**Response 4:** We sincerely apologize for the spelling issues in the original text and greatly appreciate your meticulous corrections. We have revised the relevant text to ensure an accurate description. The correct statement should read: "Black dots indicate the carbon emission reduction of GV, and the pink line represents the LLE." We have also checked and amended similar issues throughout the text to ensure the accuracy of all wording.

**Comment 5:** *While the study is focused on China’s auto market, it would be helpful to include a discussion of how the findings might apply or differ in other countries or regions, given that the NEV transition is a global phenomenon. This should be considered in the next research.*

**Response 5:** We greatly appreciate your suggestions. As you pointed out, the importance of extending the research findings to other countries or regions is indeed crucial. We have included this aspect in the revised manuscript and plan to further explore this direction in future research. The relevant content is provided below for your quick reference.

*Although this study concentrates on China's automotive market, it is essential to acknowledge the global shift towards new energy in the automotive industry. Therefore, our future research will aim to explore a more universally applicable approach to facilitate the new energy transformation of the automotive sector across various countries and regions worldwide.*

**Comment 6:** *Equations should be followed by punctuation marks and the word “where” should not be capitalized after equations, as it goes along with them here.*

**Response 6:** We greatly appreciate your meticulous corrections. The issues you pointed out regarding punctuation after equations and the capitalization of the word "where" indeed needed to be addressed. We have revised the relevant text to ensure the accurate use of punctuation and have changed "where" to lowercase to comply with academic writing standards.

**Comment 7:** *In the abstract, the sentence “with increase of production” should be revised to “with the increase in production.”*

**Response 7:** We sincerely apologize for the grammatical issues in the original text and greatly appreciate your careful corrections. We have revised the relevant sentences in the abstract to ensure grammatical accuracy and improve the fluidity of expression.

**Comment 8:** *Inconsistent spelling of terms like “behavior” and “behaviour”. In the abstract you write “we explored dynamic behaviours, …”*

**Response 8:** We greatly appreciate your meticulous corrections and sincerely apologize for the spelling issues in the original text. The inconsistency in the spelling of terms such as "behavior" and "behaviour" that you pointed out indeed needed to be addressed. We have standardized the spelling throughout the text to ensure consistency. In the abstract, we will make the corresponding change to "we explored dynamic behaviors, ...".

We appreciate your consideration of our revised manuscript and look forward to your feedback. Should you require any additional information or have any points that need clarification, please do not hesitate to contact me. During this revision process, we have thoroughly polished the paper to ensure the precision of the arguments and the clarity of the expression.

Thank you for your time and consideration.

Sincerely Yours,

[Qiong Wu]

[School of Economics and Management, Shenyang Aerospace University]

[Shenyang Aerospace University, Shenyang, 110136, China]

[+8618447071705]

[18447071705@163.com]
