# Supplementary material for: Research on complex dynamic behavior of automakers based on complex system theory
Source: PLoS One. 2024 Dec 6;19(12):e0314899. doi: 10.1371/journal.pone.0314899 (PMC11623478; doi:10.1371/journal.pone.0314899)
Supplement: Attachment — Submitted filename: Response to Reviewers.docx [file pone.0314899.s003.docx]

Dear Editor and reviewers of PLOS ONE：

I am writing to submit the revised version of our manuscript titled "**Research on complex dynamic behavior of automakers based on complex system theory**". We would like to express our sincere gratitude for the valuable time and constructive suggestions you and the reviewers have provided on our previously submitted manuscript.

We have carefully considered the feedback from you and the reviewers, which has been instrumental to our research and has greatly improved the quality of our paper. To assist with your review and that of the reviewers, we have highlighted all the added and revised sections in green in the "Revised Manuscript with Track Changes" document. Based on this valuable input, we have made the following enhancements to the manuscript:

1. We have updated the funding information in the paper. We noted that the originally listed funding project number 71702112, led by our team in 2023, has been successfully completed this year. Therefore, we would like to request its removal from the funding section of the paper to ensure that all listed information is current and accurate. Additionally, we wish to retain the funding project number L19CGL008, which is currently under the supervision of Jinhuan Tang, as it continues to support our research efforts.

2. We would like to clarify the role of the funder in this study. The funder had no involvement in the study design, data collection and analysis, decision to publish, or manuscript preparation. Therefore, we state the following: "The funders had no role in study design, data collection and analysis, decision to publish, or preparation of the manuscript."

3. Following your advice, we conducted a thorough review of the references in our paper and have confirmed that none of the cited articles have been retracted. Additionally, we have made the necessary adjustments to the formatting of the term "New media" in our references.

4. We first optimized the images using the Preflight Analysis and Conversion Engine (PACE) to ensure they meet the journal's formatting and quality standards. Additionally, we have uploaded each revised image as a separate file to facilitate easier access and review for you and the reviewers.

5. Since the reviewers did not provide specific suggestions for revision in this round, we have not prepared individual responses to the reviewers' comments.

We appreciate your consideration of our revised manuscript and look forward to your feedback. Should you require any additional information or have any points that need clarification, please do not hesitate to contact me.

Thank you once again for your assistance.

Sincerely Yours,

[Qiong Wu]

[School of Economics and Management, Shenyang Aerospace University]

[Shenyang Aerospace University, Shenyang, 110136, China]

[+8618447071705]

[18447071705@163.com]
